# Mutations in alpha‐B‐crystallin cause autosomal dominant axonal Charcot–Marie–Tooth disease with congenital cataracts

**DOI:** 10.1111/ene.16063

**Published:** 2023-09-29

**Authors:** Andrea Cortese, Riccardo Currò, Riccardo Ronco, Julian Blake, Alex M. Rossor, Enrico Bugiardini, Matilde Laurà, Tom Warner, Tarek Yousry, Roy Poh, James Polke, Adriana Rebelo, Maike F. Dohrn, Mario Saporta, Henry Houlden, Stephan Zuchner, Mary M. Reilly

**Affiliations:** ^1^ Department of Neuromuscolar Diseases UCL Queen Square Institute of Neurology London UK; ^2^ Department of Brain and Behavioral Sciences University of Pavia Pavia Italy; ^3^ Department of Clinical Neurophysiology Norfolk and Norwich University Hospital Norwich UK; ^4^ Neurogenetics Unit National Hospital for Neurology and Neurosurgery London UK; ^5^ Dr John T. Macdonald Foundation Department of Human Genetics and John P. Hussman Institute for Human Genomics University of Miami Miller School of Medicine Miami Florida USA; ^6^ Department of Neurology Medical Faculty of the RWTH Aachen University Hospital Aachen Germany

**Keywords:** cataracts neuropathy, Charcot‐Marie‐Tooth, CRYAB, myopathy

## Abstract

**Background and purpose:**

Mutations in the alpha‐B‐crystallin (*CRYAB*) gene have initially been associated with myofibrillar myopathy, dilated cardiomyopathy and cataracts. For the first time, peripheral neuropathy is reported here as a novel phenotype associated with CRYAB.

**Methods:**

Whole‐exome sequencing was performed in two unrelated families with genetically unsolved axonal Charcot–Marie–Tooth disease (CMT2), assessing clinical, neurophysiological and radiological features.

**Results:**

The pathogenic *CRYAB* variant c.358A>G;p.Arg120Gly was segregated in all affected patients from two unrelated families. The disease presented as late onset CMT2 (onset over 40 years) with distal sensory and motor impairment and congenital cataracts. Muscle involvement was probably associated in cases showing mild axial and diaphragmatic weakness. In all cases, nerve conduction studies demonstrated the presence of an axonal sensorimotor neuropathy along with chronic neurogenic changes on needle examination.

**Discussion:**

In cases with late onset autosomal dominant CMT2 and congenital cataracts, it is recommended that *CRYAB* is considered for genetic testing. The identification of *CRYAB* mutations causing CMT2 further supports a continuous spectrum of expressivity, from myopathic to neuropathic and mixed forms, of a growing number of genes involved in protein degradation and chaperone‐assisted autophagy.

## INTRODUCTION

Mutations in the alpha‐B‐crystallin (*CRYAB*) gene have been associated with myofibrillar myopathy, dilated cardiomyopathy and cataracts [1, 2]. CRYAB, also known as HSPB5, is a member of a family of 10 small heat‐shock proteins that are molecularly defined by the presence of a highly conserved alpha‐B‐crystallin domain and are functionally involved in chaperone‐mediated autophagy [3]. Of interest, mutations in two other members of this family, HSBP1 and HSPB8, originally described to cause a distal hereditary motor neuropathy have more recently been found in patients with a combined motor neuropathy and myofibrillar myopathy [4, 5, 6]. However, peripheral neuropathy has never been reported to be a feature of CRYAB‐related disease.

**FIGURE 1:**
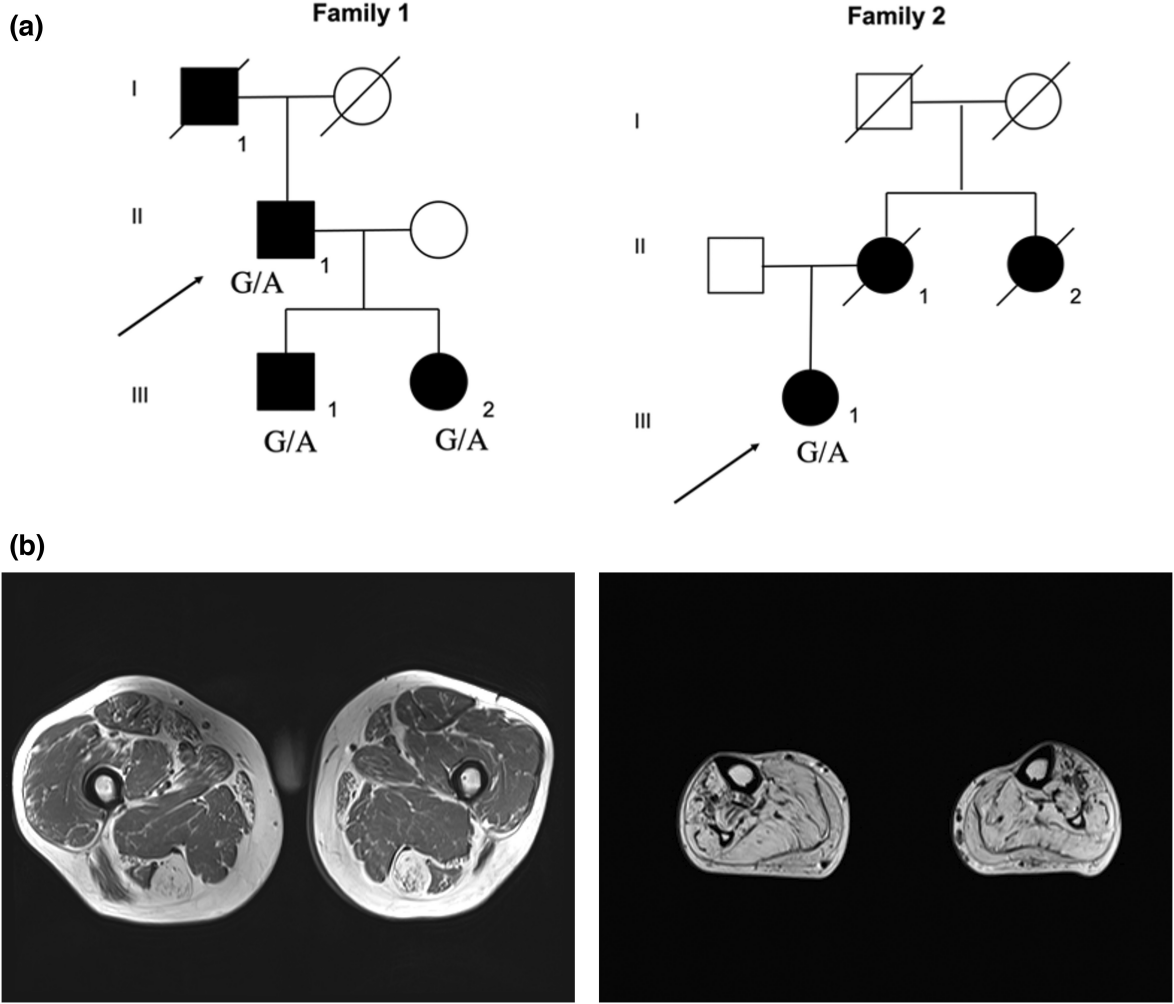
(a) Pedigree of two families with autosomal dominant CMT2 and congenital cataracts carrying the c.358A>G;p.Arg120Gly mutation in the *CRYAB* gene. The black arrow indicates the index cases. (b) Lower limb MRI of the thigh (right image) and leg (left image) muscles showing selective complete fatty involvement of the semitendinosus leg muscles (Mercuri grade 4) and mild fatty replacement of vastus lateralis, rectus femoris, gracilis and sartorius muscles of the thigh (left image, thighs).

Here, four cases with late‐onset Charcot–Marie–Tooth disease (CMT2) and congenital cataracts from two unrelated families carrying the same pathogenic c.358A>G;p.Arg120Gly mutation in *CRYAB* are described (Figure 1a and Table 1).

**TABLE 1:** Clinical and neurophysiological features of patients with CRYAB neuropathy.

	Family 1 I‐1	Family 1 II‐1	Family 1 III‐1	Family 1 III‐2	Family 2 III‐1
Current age (decade), gender	Deceased, M	Mid 60s, M	Early 30s, M	Late 20s, F	Mid 60s, F
Development	Normal	Normal	Normal	Normal	Normal
Past medical history	Caratact	Congenital cataract	Congenital cataract	Congenital cataract	Congenital cataract, bilateral hip displasia
Age of neurological onset	50s	40	–	–	Mid 40s
Symptom at onset	Walking difficulties	Walking difficultes	–	–	Walking difficulties
Neurologic examination					
Gait	High stepping gait	High stepping gait	Normal	Normal	
Weakness					
UL proximal/distal	Mild/moderate	Normal/mild	Normal	Normal	Normal/mild
LL proximal/distal	Moderate/severe	Mild/severe	Normal	Normal	Normal/mild
Sensation					
Superficial	Reduced to the ankles	Normal	Normal	Normal	Reduced to mid foot
Vibration	Reduced to costal margin	Reduced to the knees	Normal	Normal	Reduced to the knees
Reflexes					
Upper limbs	Normal	Normal	Normal	Normal	Normal
Lower limbs	Absent	Absent	Reduced	Normal	Absent
Associated features					
Congenital cataract	Yes	Yes	Yes	Yes	Yes
Possible myopathic features	Abdominal and diaphragmatic weakness	Diaphragmatic weakness	Diaphragmatic weakness (mild)		None
Heart involvement	Absent	Absent	Absent	Absent	Not tested (asymptomatic)
Neurophysiology					
Motor NCS					
Ulnar					
Amplitude mV (n.v. > 8)	4	3.8	5.5	7.9	6.8
Velocity m/s (n.v. > 50)	50	48	48	58	54
Peroneal					
Amplitude mV (n.v. > 2.5)	Not recordable	1.6	1.6	4.1	Not recordable
Velocity m/s (n.v. > 40)		48	48	48	
Sensory NCS					
Ulnar					
Amplitude μV (n.v. > 5)	Not available	2	6	25	Not recordable
Velocity m/s (n.v. > 50)		42	46	48	
Sural					
Amplitude μV (n.v. > 5)	Not available	1	Not recordable	13	Not recordable
Velocity m/s (n.v. > 40)		37		36	
Needle EMG	Neurogenic	Neurogenic	Normal	Normal	Neurogenic

Abbreviation: EMG, electromyography; LL, lower limb; NCS, nerve conduction studies; n.v., normal values; UL, upper limb.

## CASE DESCRIPTION

In family 1, originally from the UK, the index case II‐1 presented in his 40s with progressive foot drop and falls. He had a normal birth and milestones, was very good at sports and had no symptoms earlier in life. His past medical history was uneventful except for bilateral congenital cataracts, which did not need surgery. Neurological examination in his early 50s showed a high stepping gait. He could not stand on either heels or toes. Muscle strength was mildly reduced in the distal upper limb muscles and markedly reduced in the distal lower limb muscles. There was paradoxical breathing due to diaphragm weakness. Reflexes were present in the upper limbs but absent in the lower limbs. Vibration was reduced distally in the lower limbs.

Nerve conduction studies showed changes compatible with a motor and sensory axonal neuropathy, with distal predominant chronic neurogenic changes found on needle electromyography (EMG).

Magnetic resonance imaging (MRI) of the lower limbs showed widespread atrophy and severe fat replacement of the semitendinosus leg muscles and mild fat replacement of the quadriceps, gracilis and sartorius muscles of the thigh, as previously reported in CRYAB‐related myopathy [7] (Figure 1b). There was no evidence of cardiomyopathy on echocardiogram. A respiratory function test in the sitting position showed a reduced forced expiratory volume (FEV) of 1.92 L (56% predicted) and a reduced forced vital capacity (FVC) of 2.42 L (56% predicted), whilst a sleep study showed mild hypoventilation. Creatine kinase levels ranged from 150 to 352 IU/L (normal value 38–204).

Ten years later, the neuropathy had moderately progressed and he needed two crutches to walk. He was also noticed to have developed mild parkinsonism with right‐sided predominant tremor and rigidity. Brain MRI was performed and was normal, whilst a DAT scan showed impaired presynaptic dopaminergic transporter activity in both putamina in keeping with idiopathic Parkinson's disease. He was started on levodopa with only partial response.

In his family, both of his children had congenital cataracts which were operated at the age of 3 months in III‐1 but did not require surgery in III‐2. Neurological examination in III‐1, in his early 30s, was normal apart from reduced ankle reflexes. Nerve conduction studies showed the presence of a sensory and motor axonal neuropathy. III‐2, now in his late 20s, did not show any clinical or neurophysiological signs of neuropathy. A respiratory function test in III‐1 showed mild reduction(~80%) of FEV1 and FVC, whilst they were normal in III‐2.

The paternal grandfather, I‐1, had also been diagnosed with axonal neuropathy and congenital cataracts, with onset of progressive walking difficulties and high stepping gait in his fifth decade, followed by abdominal weakness, with difficulties pulling himself up from the recumbent position and diaphragmatic weakness. He died of cancer in his mid‐50s.

A next generation sequencing panel encompassing genes associated with CMT2/intermediate was performed in II‐1 but did not disclose any known cause of neuropathy. Therefore, focused exome sequencing was performed, which identified the heterozygous c.358A>G;p.Arg120Gly mutation in *CRYAB*. The mutation, which is absent from control databases, segregated with disease in the family, being also present in III‐1 and III‐2. DNA from I‐1 was not available for testing. The mutation maps to the highly conserved alpha‐crystallin domain and has been previously reported as pathogenic in multiple families.

In family 2, whose ancestors can be tracked back to Austria, the index case (III‐1) had normal birth and development and was very active and good at sports until her late 30s. Since her mid 40s she noticed some walking difficulties, which slowly progressed over the years together with imbalance and infrequent falls, impaired hand dexterity and occasional tingling in her feet. Past medical history was remarkable for the presence of bilateral congenital cataract. Examination in her mid 60s showed distal atrophy and mild weakness in the four limbs (Video S1). Reflexes were present in the upper limbs but absent in the lower limbs. Walking was independent but cautious, and she could not stand on heels or toes. Sensation to pinprick was reduced in the lower limbs to mid foot level, whilst vibration was reduced to the knees. Nerve conduction studies were performed and showed reduced motor and sensory action potentials in all four limbs, along with chronic neurogenic changes on EMG examination in distal and proximal muscles.

In her family, the patient's mother and maternal aunt were also affected by a neuromuscular condition with progressive walking difficulties and foot drop since their fifth decade of life. Notably, II‐1 died of an unspecified heart condition in his 80s. Both II‐1 and II‐2 were deceased at the time of the study and no DNA was available for testing.

Whole‐exome sequencing analysis was performed on the GENESIS platform [8] in III‐1 and showed the presence of the same pathogenic c.358A>G;p.Arg120Gly mutation in *CRYAB*.

## DISCUSSION

Our report expands the phenotype of *CRYAB*‐related disease to encompass CMT2.

CRYAB is a major structural protein of the lens, but is also ubiquitously expressed, including in skeletal and cardiac muscle as well as in peripheral nerves, where it acts as a stress‐inducible multifunctional chaperone [3]. The Arg120Gly mutation in *CRYAB* was first identified in a French family with dominantly inherited myofibrillar myopathy, hypertrophic cardiomyopathy and cataracts. Since then, the same variant has been reported in multiple families, and numerous other missense and nonsense mutations in *CRYAB* have been identified and are reviewed in Sarparanta et al. [3]. Based on these reports, dominant CRYAB‐related disease shows a wide phenotypic variability from isolated cardiomyopathy, distal myopathy or congenital cataracts to a complex multisystem disorder. Although neurogenic changes on EMG were previously observed in a few cases [9], a sensory and motor axonal neuropathy has not been previously reported as part of this condition.

The cases reported here showed definite clinical and neurophysiology features consistent with a progressive length‐dependent axonal sensory and motor neuropathy and patients were accordingly diagnosed as having CMT2. Congenital cataracts were identified in all examined cases. The presence of mild diaphragmatic and abdominal weakness in some cases may be attributed to a concurrent myopathic process, although a muscle biopsy was not performed to confirm it. The association of the same mutation with different phenotypes suggests that additional modifiers, including genetic ones, may play a role in determining the specific organ involvement. Of note, the index case II‐1 of family 1 also developed parkinsonism. Although this is more likely to be unrelated to his underlying genetic condition, it is worth noting that CRYAB was previously shown to be highly expressed in the substantia nigra in patients with Parkinson's disease, where it participates in the glial pathology during dopaminergic neuron degeneration [10].

In conclusion, the identification of *CRYAB* mutations causing CMT2 further supports a continuous spectrum of expressivity, from myopathic to neuropathic and mixed forms, of a growing number of genes involved in protein degradation and chaperone‐assisted autophagy, including *HSPB1*, *HSPB8*, *DNAJB2* and *BAG3* [4, 5, 11].

Mutations in *CRYAB* should be suspected in cases with late onset CMT2 especially in the presence of congenital cataracts. Although the presence of proximal, diaphragmatic and abdominal weakness can further support its diagnosis, their absence does not exclude it.

The identification of mutations in *CRYAB* in patients with CMT also has important clinical implications. Regular cardiac and respiratory monitoring is advised in CRYAB‐related disease, in order to identify and possibly prevent life‐threatening cardiac complications.

## AUTHOR CONTRIBUTIONS

Cortese A: Conceptualization; investigation; funding acquisition; data curation; supervision; writing—review and editing; writing—original draft; methodology. Currò R: Data curation. Ronco R: Data curation. Blake J: Data curation. Rossor AM: Data curation. Bugiardini E: Data curation. Laurà’ M: Data curation. Warner T: Data curation. Yousry T: Data curation. Poh R: Data curation. Polke J: Data curation. Rebelo A: Data curation. Dohrn MF: Data curation. Saporta M: Data curation. Houlden H: Conceptualization; investigation; supervision; data curation; funding acquisition. Zuchner S: Conceptualization; investigation; funding acquisition; data curation; supervision. Reilly MM: Conceptualization; investigation; funding acquisition; writing—original draft; data curation; supervision.

## CONFLICT OF INTEREST STATEMENT

Authors report no competing interests.

## ETHICS STATEMENT

Participant data were collected in line with the ethically approved study ‘Charcot−Marie−Tooth Disease and Related Disorders: A Natural History Study’, reviewed by the London Queen Square Research Ethics Committee.

## Supporting information


Video S1.

Video Caption

## Data Availability

The data that support the findings of this study are available on request from the corresponding author. The data are not publicly available due to privacy or ethical restrictions.
