# Supplementary material for: Mutations in alpha‐B‐crystallin cause autosomal dominant axonal Charcot–Marie–Tooth disease with congenital cataracts
Source: Eur J Neurol. 2023 Sep 29;31(1):e16063. doi: 10.1111/ene.16063 (PMC10872581; doi:10.1111/ene.16063)
Supplement: Supplementary file 2 — Video Caption [file ENE-31-e16063-s001.docx]

Caption: Neurological examination of family 2 case III-1
